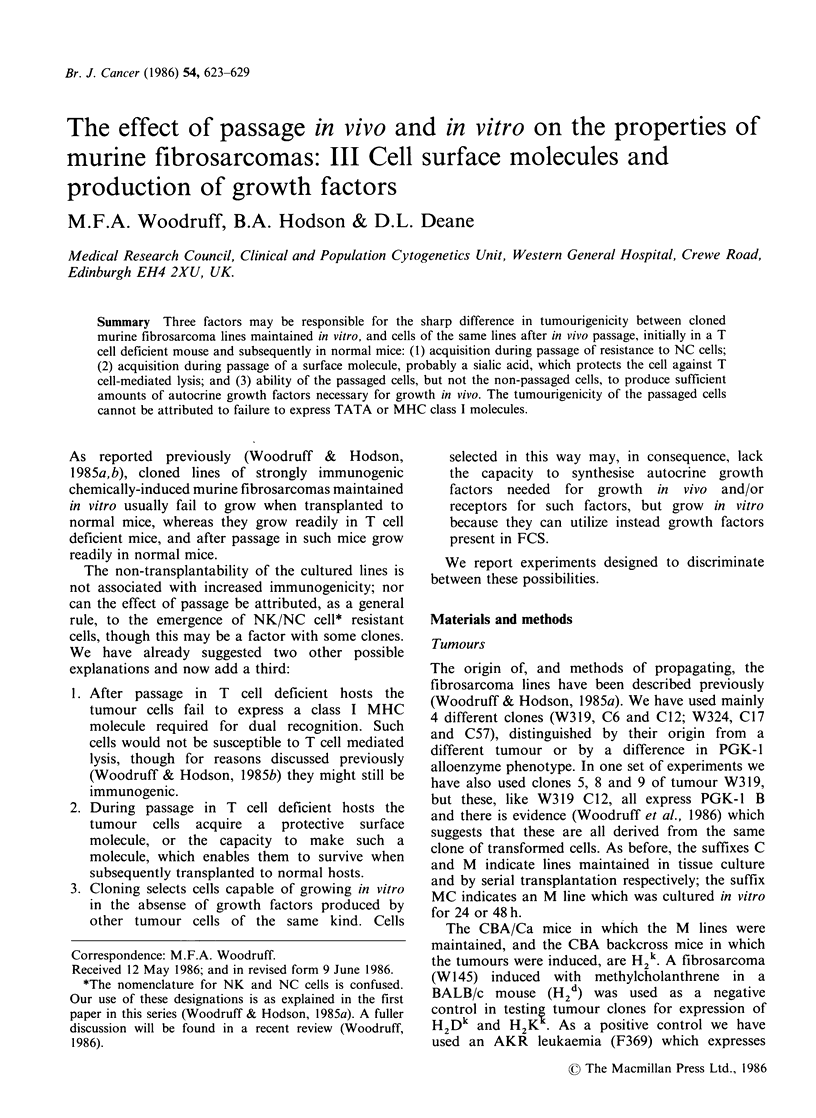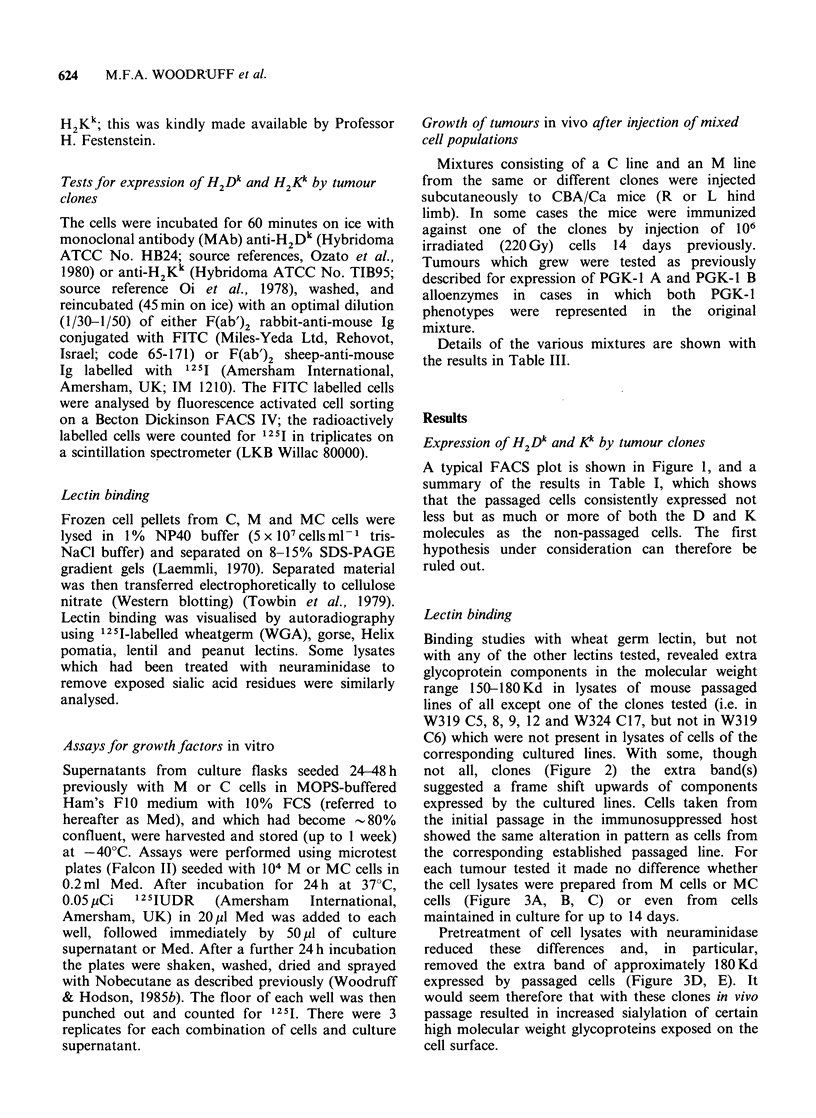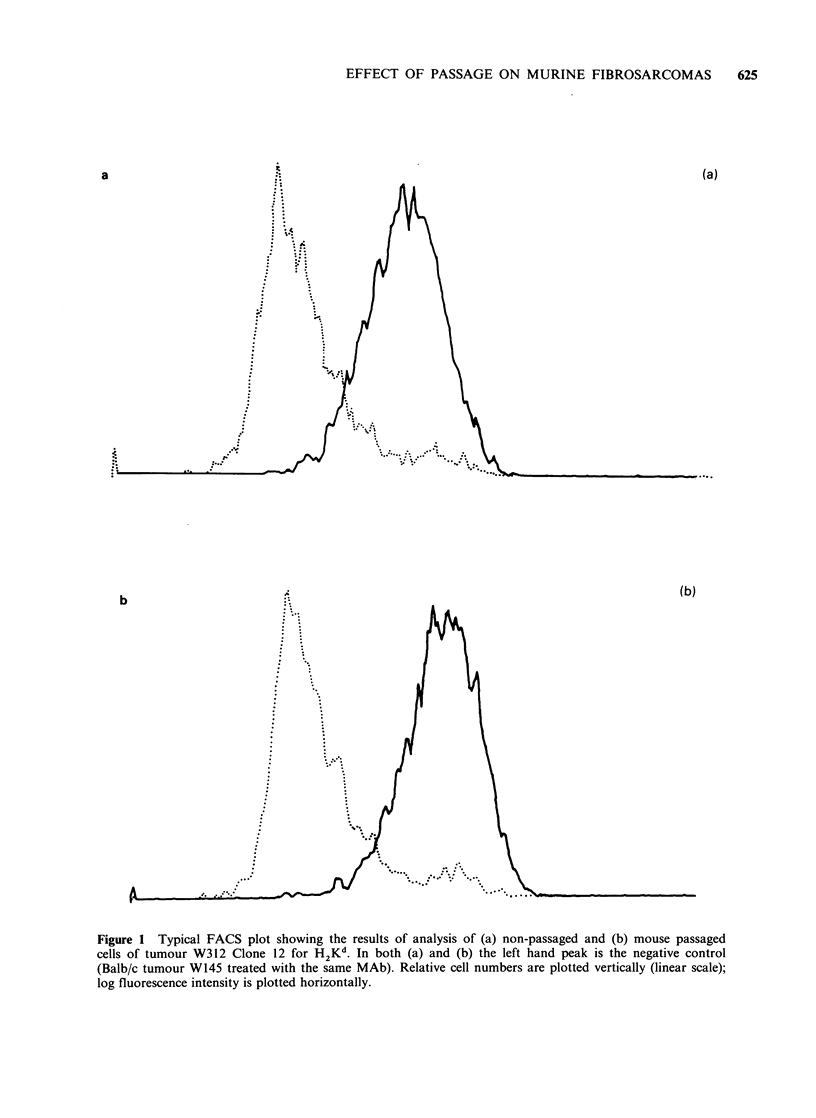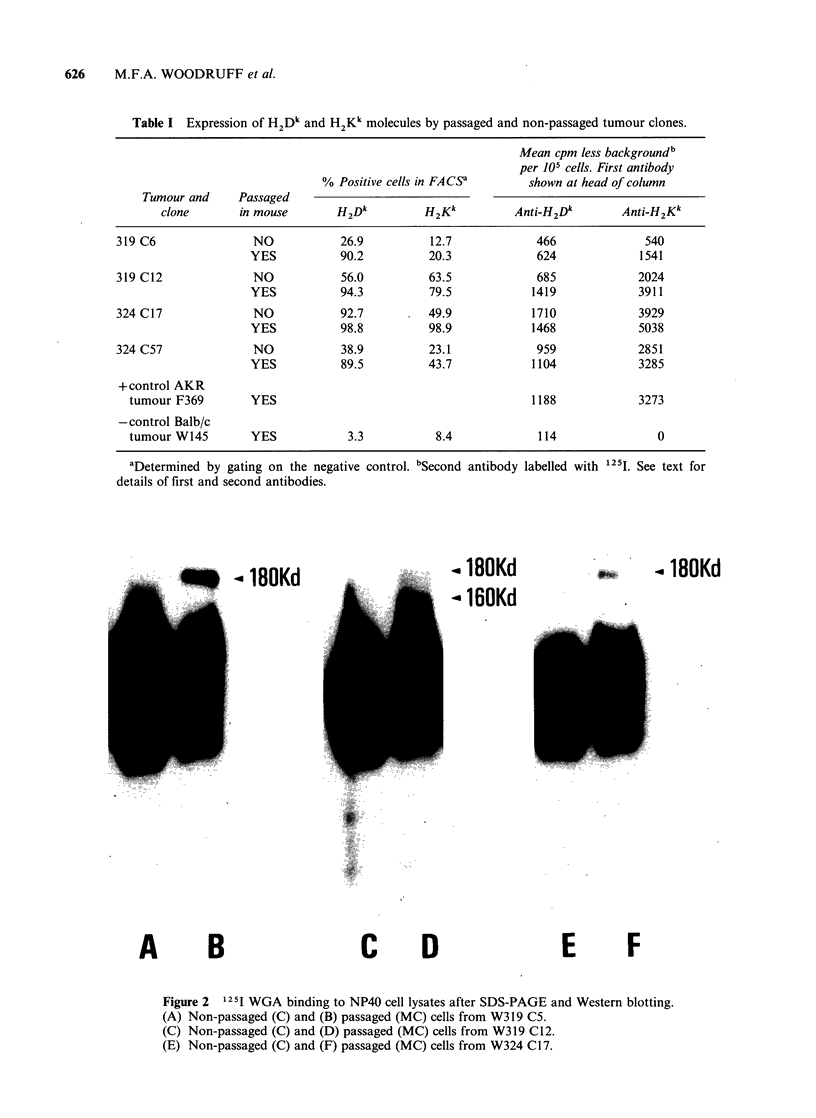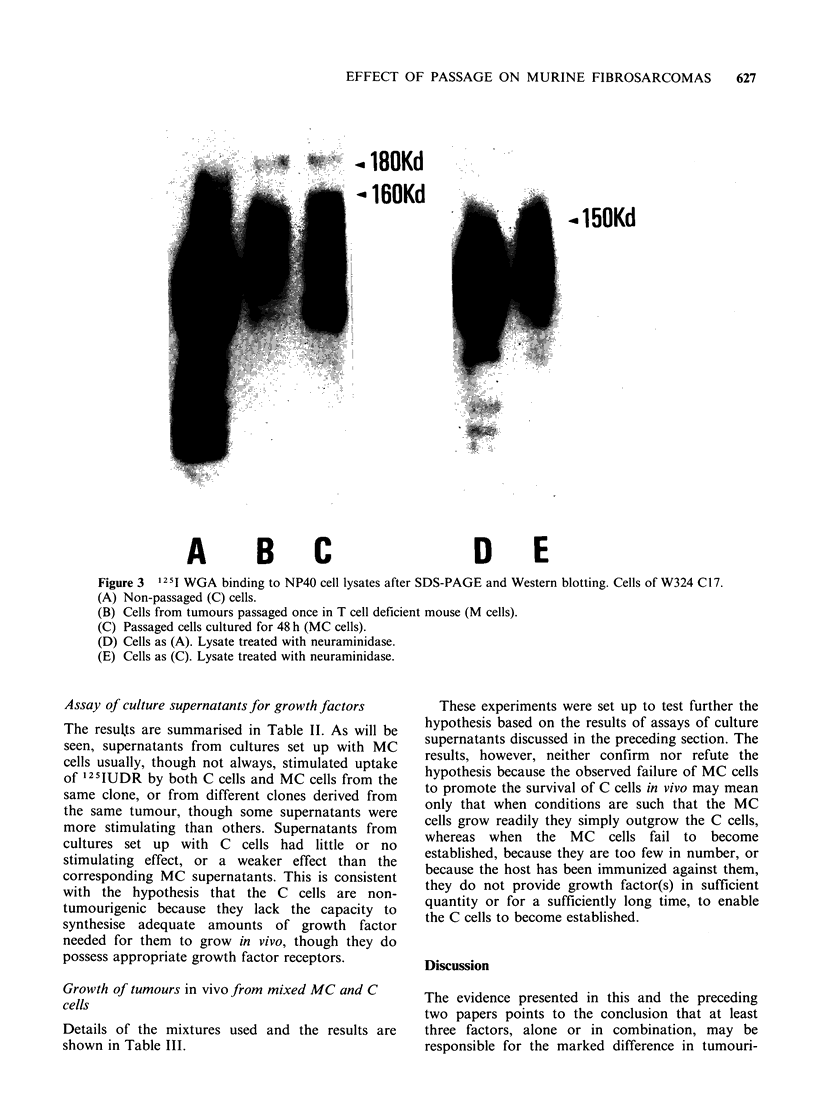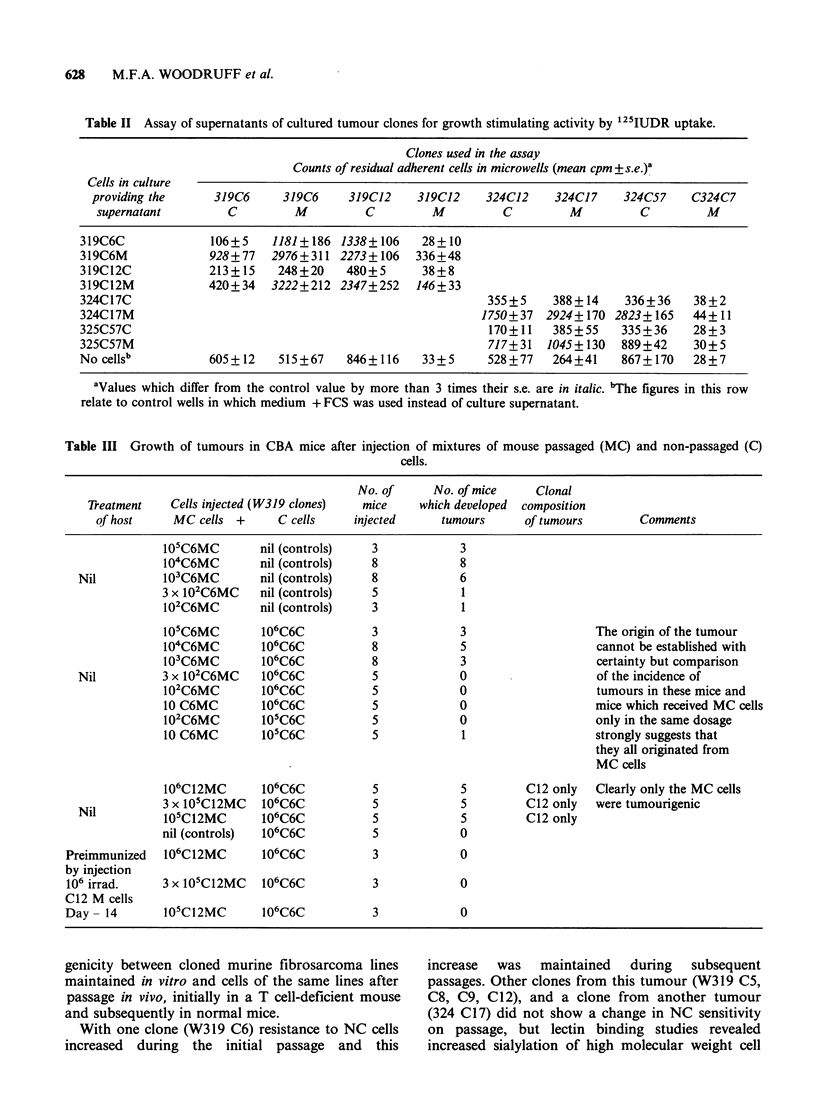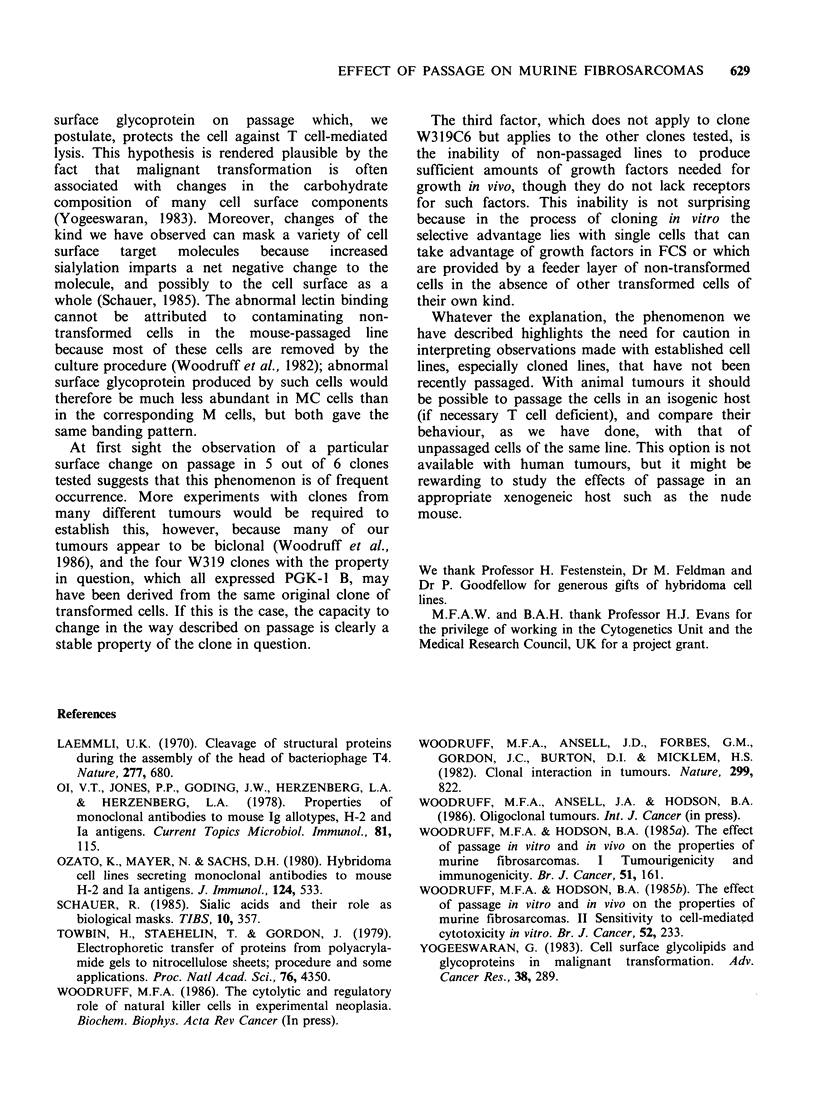# The effect of passage in vivo and in vitro on the properties of murine fibrosarcomas: III. Cell surface molecules and production of growth factors.

**DOI:** 10.1038/bjc.1986.218

**Published:** 1986-10

**Authors:** M. F. Woodruff, B. A. Hodson, D. L. Deane

## Abstract

**Images:**


*Br. J. Cancer* (1986) **54**, 623–629

### The effect of passage *in vivo* and *in vitro* on the properties of murine fibrosarcomas: III Cell surface molecules and production of growth factors

M.F.A. Woodruff, B.A. Hodson & D.L. Deane

*Medical Research Council, Clinical and Population Cytogenetics Unit, Western General Hospital, Crewe Road, Edinburgh EH4 2XU, UK.*

**Summary** Three factors may be responsible for the sharp difference in tumourigenicity between cloned murine fibrosarcoma lines maintained *in vitro*, and cells of the same lines after *in vivo* passage, initially in a T cell deficient mouse and subsequently in normal mice: (1) acquisition during passage of resistance to NC cells; (2) acquisition during passage of a surface molecule, probably a sialic acid, which protects the cell against T cell-mediated lysis; and (3) ability of the passaged cells, but not the non-passaged cells, to produce sufficient amounts of autocrine growth factors necessary for growth *in vivo*. The tumourigenicity of the passaged cells cannot be attributed to failure to express TATA or MHC class I molecules.

As reported previously (Woodruff & Hodson, 1985*a,b*), cloned lines of strongly immunogenic chemically-induced murine fibrosarcomas maintained *in vitro* usually fail to grow when transplanted to normal mice, whereas they grow readily in T cell deficient mice, and after passage in such mice grow readily in normal mice.

The non-transplantability of the cultured lines is not associated with increased immunogenicity; nor can the effect of passage be attributed, as a general rule, to the emergence of NK/NC cell* resistant cells, though this may be a factor with some clones. We have already suggested two other possible explanations and now add a third:

1. After passage in T cell deficient hosts the tumour cells fail to express a class I MHC molecule required for dual recognition. Such cells would not be susceptible to T cell mediated lysis, though for reasons discussed previously (Woodruff & Hodson, 1985*b*) they might still be immunogenic.
2. During passage in T cell deficient hosts the tumour cells acquire a protective surface molecule, or the capacity to make such a molecule, which enables them to survive when subsequently transplanted to normal hosts.
3. Cloning selects cells capable of growing *in vitro* in the absense of growth factors produced by other tumour cells of the same kind. Cells selected in this way may, in consequence, lack the capacity to synthesise autocrine growth factors needed for growth *in vivo* and/or receptors for such factors, but grow *in vitro* because they can utilize instead growth factors present in FCS.

We report experiments designed to discriminate between these possibilities.

### Materials and methods

### *Tumours*

The origin of, and methods of propagating, the fibrosarcoma lines have been described previously (Woodruff & Hodson, 1985*a*). We have used mainly 4 different clones (W319, C6 and C12; W324, C17 and C57), distinguished by their origin from a different tumour or by a difference in PGK-1 alloenzyme phenotype. In one set of experiments we have also used clones 5, 8 and 9 of tumour W319, but these, like W319 C12, all express PGK-1 B and there is evidence (Woodruff *et al.*, 1986) which suggests that these are all derived from the same clone of transformed cells. As before, the suffixes C and M indicate lines maintained in tissue culture and by serial transplantation respectively; the suffix MC indicates an M line which was cultured *in vitro* for 24 or 48 h.

The CBA/Ca mice in which the M lines were maintained, and the CBA backcross mice in which the tumours were induced, are $H_2{}^k$. A fibrosarcoma (W145) induced with methylcholanthrene in a BALB/c mouse ($H_2{}^d$) was used as a negative control in testing tumour clones for expression of $H_2D^k$ and $H_2K^k$. As a positive control we have used an AKR leukaemia (F369) which expresses

Correspondence: M.F.A. Woodruff.
Received 12 May 1986; and in revised form 9 June 1986.

*The nomenclature for NK and NC cells is confused. Our use of these designations is as explained in the first paper in this series (Woodruff & Hodson, 1985*a*). A fuller discussion will be found in a recent review (Woodruff, 1986).

© The Macmillan Press Ltd., 1986

$H_2K^k$; this was kindly made available by Professor H. Festenstein.

*Tests for expression of $H_2D^k$ and $H_2K^k$ by tumour clones*

The cells were incubated for 60 minutes on ice with monoclonal antibody (MAb) anti-$H_2D^k$ (Hybridoma ATCC No. HB24; source references, Ozato *et al.*, 1980) or anti-$H_2K^k$ (Hybridoma ATCC No. TIB95; source reference Oi *et al.*, 1978), washed, and reincubated (45 min on ice) with an optimal dilution (1/30–1/50) of either $F(ab')_2$ rabbit-anti-mouse Ig conjugated with FITC (Miles-Yeda Ltd, Rehovot, Israel; code 65-171) or $F(ab')_2$ sheep-anti-mouse Ig labelled with $^{125}I$ (Amersham International, Amersham, UK; IM 1210). The FITC labelled cells were analysed by fluorescence activated cell sorting on a Becton Dickinson FACS IV; the radioactively labelled cells were counted for $^{125}I$ in triplicates on a scintillation spectrometer (LKB Willac 80000).

*Lectin binding*

Frozen cell pellets from C, M and MC cells were lysed in 1% NP40 buffer ($5 \times 10^7$ cells ml$^{-1}$ tris-NaCl buffer) and separated on 8–15% SDS-PAGE gradient gels (Laemmli, 1970). Separated material was then transferred electrophoretically to cellulose nitrate (Western blotting) (Towbin *et al.*, 1979). Lectin binding was visualised by autoradiography using $^{125}I$-labelled wheatgerm (WGA), gorse, Helix pomatia, lentil and peanut lectins. Some lysates which had been treated with neuraminidase to remove exposed sialic acid residues were similarly analysed.

*Assays for growth factors* in vitro

Supernatants from culture flasks seeded 24–48 h previously with M or C cells in MOPS-buffered Ham's F10 medium with 10% FCS (referred to hereafter as Med), and which had become ~80% confluent, were harvested and stored (up to 1 week) at −40°C. Assays were performed using microtest plates (Falcon II) seeded with $10^4$ M or MC cells in 0.2 ml Med. After incubation for 24 h at 37°C, 0.05 $\mu$Ci $^{125}$IUDR (Amersham International, Amersham, UK) in 20 $\mu$l Med was added to each well, followed immediately by 50 $\mu$l of culture supernatant or Med. After a further 24 h incubation the plates were shaken, washed, dried and sprayed with Nobecutane as described previously (Woodruff & Hodson, 1985*b*). The floor of each well was then punched out and counted for $^{125}I$. There were 3 replicates for each combination of cells and culture supernatant.

*Growth of tumours* in vivo *after injection of mixed cell populations*

Mixtures consisting of a C line and an M line from the same or different clones were injected subcutaneously to CBA/Ca mice (R or L hind limb). In some cases the mice were immunized against one of the clones by injection of $10^6$ irradiated (220 Gy) cells 14 days previously. Tumours which grew were tested as previously described for expression of PGK-1 A and PGK-1 B alloenzymes in cases in which both PGK-1 phenotypes were represented in the original mixture.

Details of the various mixtures are shown with the results in Table III.

### Results

*Expression of $H_2D^k$ and $K^k$ by tumour clones*

A typical FACS plot is shown in Figure 1, and a summary of the results in Table I, which shows that the passaged cells consistently expressed not less but as much or more of both the D and K molecules as the non-passaged cells. The first hypothesis under consideration can therefore be ruled out.

*Lectin binding*

Binding studies with wheat germ lectin, but not with any of the other lectins tested, revealed extra glycoprotein components in the molecular weight range 150–180 Kd in lysates of mouse passaged lines of all except one of the clones tested (i.e. in W319 C5, 8, 9, 12 and W324 C17, but not in W319 C6) which were not present in lysates of cells of the corresponding cultured lines. With some, though not all, clones (Figure 2) the extra band(s) suggested a frame shift upwards of components expressed by the cultured lines. Cells taken from the initial passage in the immunosuppressed host showed the same alteration in pattern as cells from the corresponding established passaged line. For each tumour tested it made no difference whether the cell lysates were prepared from M cells or MC cells (Figure 3A, B, C) or even from cells maintained in culture for up to 14 days.

Pretreatment of cell lysates with neuraminidase reduced these differences and, in particular, removed the extra band of approximately 180 Kd expressed by passaged cells (Figure 3D, E). It would seem therefore that with these clones *in vivo* passage resulted in increased sialylation of certain high molecular weight glycoproteins exposed on the cell surface.

**Figure 1** Typical FACS plot showing the results of analysis of (a) non-passaged and (b) mouse passaged cells of tumour W312 Clone 12 for $H_2K^d$. In both (a) and (b) the left hand peak is the negative control (Balb/c tumour W145 treated with the same MAb). Relative cell numbers are plotted vertically (linear scale); log fluorescence intensity is plotted horizontally.

**Table I** Expression of $H_2D^k$ and $H_2K^k$ molecules by passaged and non-passaged tumour clones.

| *Tumour and clone* | *Passaged in mouse* | *% Positive cells in FACS*[a] | | *Mean cpm less background*[b] *per 10*[5] *cells. First antibody shown at head of column* | |
|---|---|---|---|---|---|
| | | $H_2D^k$ | $H_2K^k$ | Anti-$H_2D^k$ | Anti-$H_2K^k$ |
| 319 C6 | NO | 26.9 | 12.7 | 466 | 540 |
| | YES | 90.2 | 20.3 | 624 | 1541 |
| 319 C12 | NO | 56.0 | 63.5 | 685 | 2024 |
| | YES | 94.3 | 79.5 | 1419 | 3911 |
| 324 C17 | NO | 92.7 | 49.9 | 1710 | 3929 |
| | YES | 98.8 | 98.9 | 1468 | 5038 |
| 324 C57 | NO | 38.9 | 23.1 | 959 | 2851 |
| | YES | 89.5 | 43.7 | 1104 | 3285 |
| +control AKR tumour F369 | YES | | | 1188 | 3273 |
| −control Balb/c tumour W145 | YES | 3.3 | 8.4 | 114 | 0 |

[a]Determined by gating on the negative control. [b]Second antibody labelled with $^{125}$I. See text for details of first and second antibodies.

**Figure 2** $^{125}$I WGA binding to NP40 cell lysates after SDS-PAGE and Western blotting.
(A) Non-passaged (C) and (B) passaged (MC) cells from W319 C5.
(C) Non-passaged (C) and (D) passaged (MC) cells from W319 C12.
(E) Non-passaged (C) and (F) passaged (MC) cells from W324 C17.

**Figure 3** $^{125}$I WGA binding to NP40 cell lysates after SDS-PAGE and Western blotting. Cells of W324 C17.
(A) Non-passaged (C) cells.
(B) Cells from tumours passaged once in T cell deficient mouse (M cells).
(C) Passaged cells cultured for 48 h (MC cells).
(D) Cells as (A). Lysate treated with neuraminidase.
(E) Cells as (C). Lysate treated with neuraminidase.

*Assay of culture supernatants for growth factors*

The results are summarised in Table II. As will be seen, supernatants from cultures set up with MC cells usually, though not always, stimulated uptake of $^{125}$IUDR by both C cells and MC cells from the same clone, or from different clones derived from the same tumour, though some supernatants were more stimulating than others. Supernatants from cultures set up with C cells had little or no stimulating effect, or a weaker effect than the corresponding MC supernatants. This is consistent with the hypothesis that the C cells are non-tumourigenic because they lack the capacity to synthesise adequate amounts of growth factor needed for them to grow *in vivo*, though they do possess appropriate growth factor receptors.

*Growth of tumours* in vivo *from mixed MC and C cells*

Details of the mixtures used and the results are shown in Table III.

These experiments were set up to test further the hypothesis based on the results of assays of culture supernatants discussed in the preceding section. The results, however, neither confirm nor refute the hypothesis because the observed failure of MC cells to promote the survival of C cells *in vivo* may mean only that when conditions are such that the MC cells grow readily they simply outgrow the C cells, whereas when the MC cells fail to become established, because they are too few in number, or because the host has been immunized against them, they do not provide growth factor(s) in sufficient quantity or for a sufficiently long time, to enable the C cells to become established.

### Discussion

The evidence presented in this and the preceding two papers points to the conclusion that at least three factors, alone or in combination, may be responsible for the marked difference in tumouri-

**Table II** Assay of supernatants of cultured tumour clones for growth stimulating activity by $^{125}$IUDR uptake.

| *Cells in culture providing the supernatant* | *Clones used in the assay Counts of residual adherent cells in microwells (mean cpm ± s.e.)*[a] | | | | | | | |
|---|---|---|---|---|---|---|---|---|
| | *319C6 C* | *319C6 M* | *319C12 C* | *319C12 M* | *324C12 C* | *324C17 M* | *324C57 C* | *C324C7 M* |
| 319C6C | 106±5 | *1181*±186 | *1338*±106 | 28±10 | | | | |
| 319C6M | *928*±77 | *2976*±311 | *2273*±106 | 336±48 | | | | |
| 319C12C | 213±15 | 248±20 | 480±5 | 38±8 | | | | |
| 319C12M | 420±34 | *3222*±212 | *2347*±252 | *146*±33 | | | | |
| 324C17C | | | | | 355±5 | 388±14 | 336±36 | 38±2 |
| 324C17M | | | | | *1750*±37 | *2924*±170 | *2823*±165 | 44±11 |
| 325C57C | | | | | 170±11 | 385±55 | 335±36 | 28±3 |
| 325C57M | | | | | *717*±31 | *1045*±130 | 889±42 | 30±5 |
| No cells[b] | 605±12 | 515±67 | 846±116 | 33±5 | 528±77 | 264±41 | 867±170 | 28±7 |

[a]Values which differ from the control value by more than 3 times their s.e. are *in italic*. [b]The figures in this row relate to control wells in which medium + FCS was used instead of culture supernatant.

**Table III** Growth of tumours in CBA mice after injection of mixtures of mouse passaged (MC) and non-passaged (C) cells.

| *Treatment of host* | *Cells injected (W319 clones) MC cells +* | *C cells* | *No. of mice injected* | *No. of mice which developed tumours* | *Clonal composition of tumours* | *Comments* |
|---|---|---|---|---|---|---|
| | $10^5$C6MC | nil (controls) | 3 | 3 | | |
| | $10^4$C6MC | nil (controls) | 8 | 8 | | |
| Nil | $10^3$C6MC | nil (controls) | 8 | 6 | | |
| | $3 \times 10^2$C6MC | nil (controls) | 5 | 1 | | |
| | $10^2$C6MC | nil (controls) | 3 | 1 | | |
| | $10^5$C6MC | $10^6$C6C | 3 | 3 | | The origin of the tumour |
| | $10^4$C6MC | $10^6$C6C | 8 | 5 | | cannot be established with |
| | $10^3$C6MC | $10^6$C6C | 8 | 3 | | certainty but comparison |
| Nil | $3 \times 10^2$C6MC | $10^6$C6C | 5 | 0 | | of the incidence of |
| | $10^2$C6MC | $10^6$C6C | 5 | 0 | | tumours in these mice and |
| | 10 C6MC | $10^6$C6C | 5 | 0 | | mice which received MC cells |
| | $10^2$C6MC | $10^5$C6C | 5 | 0 | | only in the same dosage |
| | 10 C6MC | $10^5$C6C | 5 | 1 | | strongly suggests that they all originated from MC cells |
| | $10^6$C12MC | $10^6$C6C | 5 | 5 | C12 only | Clearly only the MC cells |
| Nil | $3 \times 10^5$C12MC | $10^6$C6C | 5 | 5 | C12 only | were tumourigenic |
| | $10^5$C12MC | $10^6$C6C | 5 | 5 | C12 only | |
| | nil (controls) | $10^6$C6C | 5 | 0 | | |
| Preimmunized by injection | $10^6$C12MC | $10^6$C6C | 3 | 0 | | |
| $10^6$ irrad. C12 M cells | $3 \times 10^5$C12MC | $10^6$C6C | 3 | 0 | | |
| Day - 14 | $10^5$C12MC | $10^6$C6C | 3 | 0 | | |

genicity between cloned murine fibrosarcoma lines maintained *in vitro* and cells of the same lines after passage *in vivo,* initially in a T cell-deficient mouse and subsequently in normal mice.

With one clone (W319 C6) resistance to NC cells increased during the initial passage and this increase was maintained during subsequent passages. Other clones from this tumour (W319 C5, C8, C9, C12), and a clone from another tumour (324 C17) did not show a change in NC sensitivity on passage, but lectin binding studies revealed increased sialylation of high molecular weight cell surface glycoprotein on passage which, we postulate, protects the cell against T cell-mediated lysis. This hypothesis is rendered plausible by the fact that malignant transformation is often associated with changes in the carbohydrate composition of many cell surface components (Yogeeswaran, 1983). Moreover, changes of the kind we have observed can mask a variety of cell surface target molecules because increased sialylation imparts a net negative change to the molecule, and possibly to the cell surface as a whole (Schauer, 1985). The abnormal lectin binding cannot be attributed to contaminating non-transformed cells in the mouse-passaged line because most of these cells are removed by the culture procedure (Woodruff *et al.*, 1982); abnormal surface glycoprotein produced by such cells would therefore be much less abundant in MC cells than in the corresponding M cells, but both gave the same banding pattern.

At first sight the observation of a particular surface change on passage in 5 out of 6 clones tested suggests that this phenomenon is of frequent occurrence. More experiments with clones from many different tumours would be required to establish this, however, because many of our tumours appear to be biclonal (Woodruff *et al.*, 1986), and the four W319 clones with the property in question, which all expressed PGK-1 B, may have been derived from the same original clone of transformed cells. If this is the case, the capacity to change in the way described on passage is clearly a stable property of the clone in question.

The third factor, which does not apply to clone W319C6 but applies to the other clones tested, is the inability of non-passaged lines to produce sufficient amounts of growth factors needed for growth *in vivo*, though they do not lack receptors for such factors. This inability is not surprising because in the process of cloning *in vitro* the selective advantage lies with single cells that can take advantage of growth factors in FCS or which are provided by a feeder layer of non-transformed cells in the absence of other transformed cells of their own kind.

Whatever the explanation, the phenomenon we have described highlights the need for caution in interpreting observations made with established cell lines, especially cloned lines, that have not been recently passaged. With animal tumours it should be possible to passage the cells in an isogenic host (if necessary T cell deficient), and compare their behaviour, as we have done, with that of unpassaged cells of the same line. This option is not available with human tumours, but it might be rewarding to study the effects of passage in an appropriate xenogeneic host such as the nude mouse.

We thank Professor H. Festenstein, Dr M. Feldman and Dr P. Goodfellow for generous gifts of hybridoma cell lines.

M.F.A.W. and B.A.H. thank Professor H.J. Evans for the privilege of working in the Cytogenetics Unit and the Medical Research Council, UK for a project grant.

### References

LAEMMLI, U.K. (1970). Cleavage of structural proteins during the assembly of the head of bacteriophage T4. *Nature*, **277**, 680.

OI, V.T., JONES, P.P., GODING, J.W., HERZENBERG, L.A. & HERZENBERG, L.A. (1978). Properties of monoclonal antibodies to mouse Ig allotypes, H-2 and Ia antigens. *Current Topics Microbiol. Immunol.*, **81**, 115.

OZATO, K., MAYER, N. & SACHS, D.H. (1980). Hybridoma cell lines secreting monoclonal antibodies to mouse H-2 and Ia antigens. *J. Immunol.*, **124**, 533.

SCHAUER, R. (1985). Sialic acids and their role as biological masks. *TIBS*, **10**, 357.

TOWBIN, H., STAEHELIN, T. & GORDON, J. (1979). Electrophoretic transfer of proteins from polyacrylamide gels to nitrocellulose sheets; procedure and some applications. *Proc. Natl Acad. Sci.*, **76**, 4350.

WOODRUFF, M.F.A. (1986). The cytolytic and regulatory role of natural killer cells in experimental neoplasia. *Biochem. Biophys. Acta Rev Cancer* (In press).

WOODRUFF, M.F.A., ANSELL, J.D., FORBES, G.M., GORDON, J.C., BURTON, D.I. & MICKLEM, H.S. (1982). Clonal interaction in tumours. *Nature*, **299**, 822.

WOODRUFF, M.F.A., ANSELL, J.A. & HODSON, B.A. (1986). Oligoclonal tumours. *Int. J. Cancer* (in press).

WOODRUFF, M.F.A. & HODSON, B.A. (1985*a*). The effect of passage *in vitro* and *in vivo* on the properties of murine fibrosarcomas. I Tumourigenicity and immunogenicity. *Br. J. Cancer*, **51**, 161.

WOODRUFF, M.F.A. & HODSON, B.A. (1985*b*). The effect of passage *in vitro* and *in vivo* on the properties of murine fibrosarcomas. II Sensitivity to cell-mediated cytotoxicity *in vitro*. *Br. J. Cancer*, **52**, 233.

YOGEESWARAN, G. (1983). Cell surface glycolipids and glycoproteins in malignant transformation. *Adv. Cancer Res.*, **38**, 289.